# A rare case of orbital apex syndrome with herpes zoster ophthalmicus in a human immunodeficiency virus-positive patient

**DOI:** 10.4103/0301-4738.71708

**Published:** 2010

**Authors:** Rohit Saxena, Swati Phuljhele, Lalit Aalok, Ankur Sinha, Vimla Menon, Pradeep Sharma, Anant Mohan

**Affiliations:** Squint and Neuro-Ophthalmology Services, Dr. Rajendra Prasad Center for Ophthalmic Sciences, All India Institute of Medical Sciences, New Delhi, India; 1 Department of Medicine, All India Institute of Medical Sciences, New Delhi, India

**Keywords:** HIV, HZO, orbital apex syndrome

## Abstract

We report a rare instance of favorable outcome in orbital apex syndrome secondary to herpes zoster ophthalmicus (HZO) in a human immunodeficiency virus (HIV)-positive patient. The patient complained of pain and decrease in vision in one eye (20/640) for 2 weeks accompanied with swelling, inability to open eye, and rashes around the periocular area and forehead. The presence of complete ophthalmoplegia, ptosis, relative afferent pupillary defect, and anterior uveitis with decreased corneal sensation prompted a diagnosis of HZO with orbital apex syndrome. The enzyme-linked immunosorbent assay test and a low CD4 count confirmed HIV. Highly active antiretroviral therapy (HAART), systemic acyclovir, and systemic steroids were started. Visual acuity and uveitis improved within 10 days. By the end of the fourth week, ocular motility also recovered and the final visual acuity was 20/25. We highlight the role of HAART, used in conjunction with systemic steroid and acyclovir therapy, in improving the outcome.

Orbital apex syndrome is a known but rare manifestation of herpes zoster virus (HZV) infection. Treatment with systemic acyclovir and steroids usually carries good prognosis. The presence of immunosuppression, particularly infection with human immunodeficiency virus (HIV), complicates the presentation and changes the treatment strategy. The use of steroids in such a condition becomes controversial, and previously reported prognosis is poor.[1,2] In the following case, we highlight the role of highly active antiretroviral therapy (HAART) as part of treatment with the judicious use of steroids in improving the outcome in orbital apex syndrome caused by HZV with coexisting HIV infection.

## Case Report

A 29-year-old female presented with a 2-week history of decrease in vision and pain in the left eye (LE) associated with swelling and inability to open the eyelids and rashes around the left periocular area and forehead.

**Figure 1:**
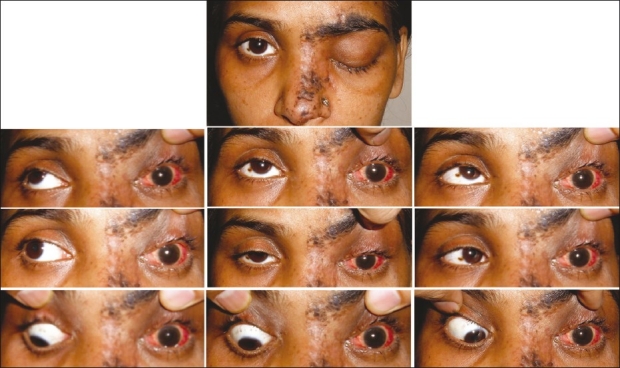
At presentation: showing vesiculopustular rashes, ptosis, and limited ocular motility

**Figure 2:**
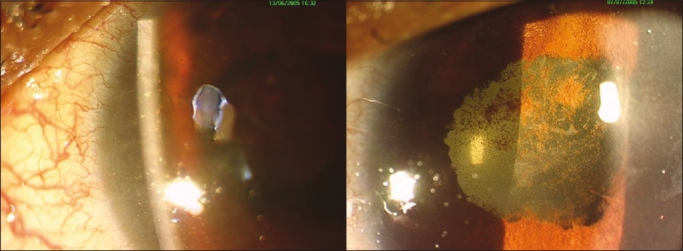
Showing the presence of old keratic precipitates and posterior synechia

On examination, resolving vesiculopustular skin lesions involved the left half of forehead and left periocular region and there existed severe ptosis and a complete limitation of ocular motility [Fig. 1]. Forced duction test was negative in all directions. Visual acuity was 20/20 in the right eye and 20/640 in the LE with accurate projection of rays in both the eyes. A slit lamp biomicroscopic examination revealed corneal edema and signs of keratouveitis in the LE (keratic precipitates, cells, and flare in the anterior chamber) and pigmentation on the lens capsule [Fig. 2]. Direct pupil response could not be assessed due to the presence of 360° posterior synechia; however, consensual light response was sluggish in the right eye. Sensations over the left half of the forehead and left cornea were impaired. Fundus examination revealed normal discs and macula in both eyes although soft exudates were present in the inferior retina of the LE. A clinical diagnosis of HZO with orbital apex syndrome of the LE was made. Due to the severity of HZO and the presence of soft exudates, a coexisting HIV infection was suspected.

**Figure 3:**
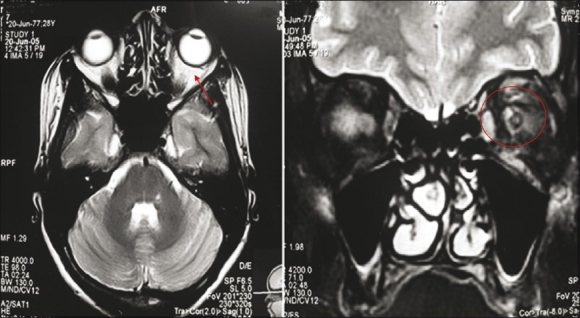
MRI: edema in the left extraocular muscle cone and retrobulbar fat extending into the orbital apex and subtle hyperintensity of the left optic nerve in the intraorbital and intracanalicular portion

Blood investigations revealed normal levels of hemoglobin, blood counts, and ESR. The enzyme-linked immunosorbent assay (ELISA) test was positive for HIV. CD4 and CD8 cell counts were 171 and 339 cells per micro liter, respectively, and the CD4/CD8 ratio was 0.5 (reversed). A magnetic resonance imaging (MRI) scan revealed edema in the left extraocular muscle cone and retrobulbar fat extending into the orbital apex along with subtle hyperintensity of the left optic nerve in the intraorbital and intracanalicular portions [Fig. 3]. The latency and amplitude of pattern visual evoked potential was normal in the right eye but was extinguished in the left eye.

**Figure 4:**
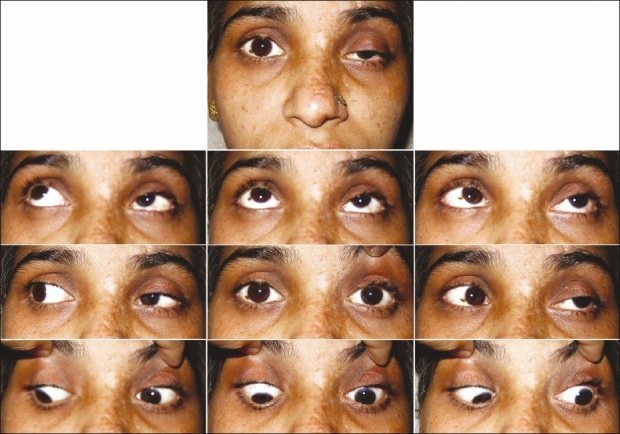
After 4 weeks of treatment: improvement of ptosis and ocular motility

The patient was unwilling for admission and initial treatment in the form of HAART and oral acyclovir (800 mg five times a day) was commenced. She presented for admission after 5 days when oral prednisolone (1 mg/kg/day) was also started after consulting a physician. Topical acyclovir eye ointment (five times a day) along with topical steroids and cycloplegics were prescribed. By the 10^th^ day, there was improvement in uveitis, and vision improved to 20/50 though ocular motility did not improve significantly. Systemic steroids were tapered over the next 10 days while HAART and systemic acyclovir were continued. At the end of 4 weeks, the vision improved to 20/25 and there was improvement in ocular motility and ptosis as well [Fig. 4].

## Discussion

HZO may be the presenting feature of AIDS-related complex and AIDS[3] and can lead to more serious sight-threatening complications in such patients.[4] In patients of HZO, coexisting HIV infection should be suspected if there is presence of peripheral retinal perivasculitis and perivascular sheathing,[5] younger age at onset, affection of multiple dermatomes, the progressive outer retinal necrosis syndrome, chronic dendritic infection, an ocular disease sine herpete, and serious neurologic disease.

Although there are several case reports of internal or external ophthalmoplegia (partial or total),[6–8] optic neuropathy in HZO is rare, with orbital apex syndrome being reported in only four cases.[9–11] We report a case of HZO with its varied ocular manifestations in a single patient. The severity of presentation in our case, which included keratitis, anterior uveitis, and total ophthalmoplegia along with optic nerve involvement, is possibly explained by the immunocompromised status owing to the coexisting HIV infection. The treatment of HZO including that of orbital apex syndrome consists of systemic antivirals and steroids[10] and the prognosis for ophthalmoplegia and optic neuropathy following HZO in immunocompetent patients is good. Coexisting HIV infection increases the severity and chronicity of complications, necessitating prolonged therapy. HZO in the presence of HIV is treated with intravenous acyclovir followed by oral acyclovir. The role of steroids for the treatment of orbital apex syndrome in immunocompromised patients is controversial. Kattah *et al*. reported two patients of HZO with orbital apex syndrome. The patient with normal immunity rapidly improved on steroid therapy; however, the patient with Hodgkin’s disease suffered a protracted course of the disease subsequently developing secondary bacterial endophthalmitis that necessitated evisceration of the eye.[9] In our case, we gave steroid therapy under the cover of HAART and systemic acyclovir and the patient’s systemic condition was closely monitored. Oral steroids were rapidly tapered over 10 days. The patient did not develop any systemic or ocular complications from steroid therapy during this period.

The available literature on HZO has not discussed the effect or role of HAART in the presence of concomitant HIV infection. However, the incidence of ocular complications of HIV infection has decreased particularly in the developed countries because of the use of HAART,[12] but the role of HAART as a part of treatment for ocular involvement in HZO with HIV is yet to be established. Reports of optic neuropathy in the pre-HAART era when treated with acyclovir with or without systemic steroids show poor prognosis.[1,2] We believe that the favorable prognosis in our case can be attributed to the prompt induction of HAART along with the judicious use of systemic steroids.

## Conclusion

A severe presentation of HZO particularly in young patients warrants an evaluation of the HIV status of the patient. Simultaneous induction of HAART and systemic acyclovir along with cautious use of steroids may have favorable prognosis.
